# Comparative study of short- and long-term outcomes of laparoscopic-assisted versus open rectal cancer resection during and after the learning curve period

**DOI:** 10.1097/MD.0000000000006909

**Published:** 2017-05-12

**Authors:** Yunhua Wu, Xuejun Sun, Jie Qi, Guangbing Wei, Feibo Cui, Qi Gao, Junhui Yu, Kai Wang, Jianbao Zheng

**Affiliations:** a Department of General Surgery, First Affiliated Hospital of Xi’an Jiaotong University; b Second Department of Cardiovascular Medicine, Shaanxi Provincial People's Hospital, Xi’an, Shaanxi, PR China.

**Keywords:** laparoscopic-assisted rectal resection, learning curve, oncological outcomes, overall survival rate, rectal cancer

## Abstract

Laparoscopic-assisted rectal resection (LAR) has been widely used to treat rectal cancer. However, it has a steep learning curve. In this study, we aimed to investigate the effects of the learning curve on the outcomes of LAR. All consecutive patients with rectal cancer undergoing LAR or open resection (OR) between 2010 and 2015 were included in this retrospective analysis. The learning curve was determined, and patients were divided into 2 phases: the learning curve and the expert period. The short-term perioperative data in the 2 phases and the long-term survival in the learning phase were compared between the LAR and OR groups. A total of 491 patients were included in this study. Inflection of the learning curve based on the operation time of LAR was at the 40th case. A total of 233 patients underwent surgery (112 LAR and 121 OR) during the learning period. In this period, LAR had a longer operation time, less blood loss, and a higher total cost (all *P* < .05). The 3-year overall survival rates between the LAR and OR groups were similar (69.74% vs 75%; *P* = .32). A total of 258 patients underwent surgery (169 LAR and 89 OR) during the expert period. Significant differences in total cost, estimated blood loss, postoperative hospital stay, and recovery of bladder and bowel functions were identified in this period (all *P* < .05). LAR during the learning period has fewer benefits in terms of postoperative recovery than OR. However, the long-term outcomes are equivalent.

## 1 Introduction

Colorectal cancer is one of the most common types of gastrointestinal tumors. It is estimated that as of January 1, 2014, there are more than 1.2 million men and women living in the United States with a history of colorectal cancer.^[1–3]^ In spite of various treatment modalities for this disease, surgical resection remains the mainstay of treatment.^[4,5]^ Since the first description of laparoscopic surgery, various randomized controlled trials have been conducted and a recent meta-analysis has acknowledged that the laparoscopic approach for the treatment of rectal cancer has advantages over conventional surgery: less blood loss, quicker recovery, less pain, shorter hospital stay, and equivalent oncological outcomes.^[6]^

However, laparoscopic-assisted rectal resection (LAR) demands a relatively long training period for surgeons to become technically expert because of the steep learning curve.^[7]^ The number of cases that a surgeon needs to operate on in order to obtain technical capability for a surgical procedure is defined as the learning curve. It reflects both the level of technical difficulty and the ability to adapt to a new technique. Based on several previous reports, the learning curve for laparoscopic colorectal resections ranges from 30 to 70 cases.^[8–11]^ However, very few reports have focused on comparison of the clinical outcomes between LAR and open resection (OR) during and after the learning curve period. The safety and clinical outcomes of LAR during the learning curve period are unclear. In the present study, we aimed to assess the learning curve using the moving average method, cumulative sum analysis (CUSUM), risk-adjusted CUSUM, and Matlab software (The MathWorks, Inc., Natick, MA) in LAR and compare the short- and long-term clinical outcomes of LAR with OR during and after the learning curve period.

## 2 Methods

### 2.1 Study design

The present retrospective study was approved by the research ethics committee of the First Affiliated Hospital of Xi’an Jiaotong University, and written informed consents were obtained from all patients.

We included all consecutive patients with rectal cancer undergoing radical rectal resection at the First Affiliated Hospital of Xi’an Jiaotong University between October 2010 and December 2015. Patients with the following criteria were excluded: in situ or metastatic disease, noncurative resection, emergency presentation, body mass index (BMI) > 35 kg/m^2^, patients with an American Society of Anesthesiologists (ASA) score of 4 or more, and patients who had preoperative radiotherapy or chemotherapy, major abdominal surgery, pregnancy, or malignant disease in the past 5 years.

### 2.2 Patients and clinical data

OR and LAR were performed by 3 stable surgical groups, respectively. Basic clinical parameters and short-term outcomes were collected from the First Affiliated Hospital of Xi’an Jiaotong University database. Common data, including age, sex, abdominal operation history, BMI, accompanied disease, the inferior average to anal margin, ASA physical status classification, tumor, lymph node, metastasis (TNM) stage, tumor diameter, histology of tumor, surgical procedures, prevented colostomy or ileostomy, lymphovascular invasion, and carcinoembryonic antigen (CEA) level, were collected. The postoperative TNM stage was determined according to the 7th edition of the American Joint Committee on Cancer staging manual. The short-term outcomes included total cost, operation time, postoperative hospital stay, blood loss, times to first soft diet and first flatus, harvested lymph nodes, distal and proximal margins, and postoperative complications.

### 2.3 Preoperative preparation, operation procedures, and postoperative management

All patients included in this study underwent preoperative laboratory examinations including tumor marker screening, coagulation test, chest X-ray, computed tomography scan of the abdomen and pelvis, endoscopy for confirmation of tumor localization, and biopsy for a clear diagnosis of rectal cancer. The patients received bowel preparation with polyethylene glycol-electrolyte solution or magnesium sulfate. Two hours before surgery, intravenous cefmetazole sodium was given as a prophylactic antibiotic.

The decision for LAR or OR was made according to the preferences of the individual surgeons, operating theater availability, and individual patient's consent. The laparoscopic-associated rectal resection was performed utilizing a medial-to-lateral approach in the majority of cases. When feasible, a standard total mesorectal excision was performed for every LAR case. Decision for conversion from LAR to OR was left to the discretion of the surgeon based on concerns regarding patient safety, technical difficulties, or intraoperative findings that suggested a laparotomy. The cases who underwent conversion were included in the laparoscopic arm of the study. But these cases were excluded from further analysis. Open procedures were performed according to the standard techniques as described previously.^[12]^ A standard D2 lymph node dissection was performed in every case of both groups according to the Guidelines of Radical Laparoscopic Colorectal Cancer Surgery (2008) established by the Study Group of Laparoscopic and Endoscopic Surgery Affiliated to the Chinese Medical Association^[11]^ and the American Joint Committee on Cancer 7th edition guidelines.^[7]^

Postoperative management was standardized. Three groups of patients receiving surgery, defined as groups A, B, and C, were supported by infusions in the very first several hours after surgery. Prophylactic antibiotics were used for 48 hours after surgery; however, if there was any indication of infection, the duration of antibiotics was prolonged. Postoperative care included chest physiotherapy, ambulation, low-flow oxygenation, and an atomizer inhaler. A clear liquid diet was supplied after the first passage of flatus. Patient-controlled anesthesia was given for pain control in the first 2 days after surgery. In addition, short-acting drugs were used according to the patient's demands. The management of patients with postoperative complications was the same in all groups. Furthermore, every patient who was pathologically diagnosed to have stage III disease was treated with adjuvant chemotherapy (5-FU and oxaliplatin [FOLFOX]) and 50-Gy radiotherapy for 6 months.

### 2.4 Follow-up

All patients were subjected to close follow-up for the first month after surgery, followed by every 3 months for the first 2 years, and every 6 months for the next 3 years. Data were collected prospectively from the time of diagnosis using a custom-written computerized database. The last follow-up date was December 2015.

### 2.5 Learning curve analysis

We selected 2 representative groups from the 3 stable surgical groups for the learning curve analysis, defined as group A and group B. The moving average method, CUSUM, and Matlab software were employed to determine the learning curve by detecting a shift in the trend of operation time as described previously.^[8–10,13–15]^

### 2.6 Statistical analysis

Statistical analysis in this study was performed using SPSS software package version 13.0 (SPSS Inc., Chicago, IL). Continuous variables were analyzed using the Student *t* test or the Mann–Whitney test. Categorical variables were analyzed using the Chi-square test or Fisher exact test. The 3-year survival ratio was calculated by the Kaplan–Meier method, and the log-rank test was used to analyze the differences. *P* < .05 was considered to be statistically significant.

## 3 Results

### 3.1 Estimation of the learning curve

**Figure 1:**
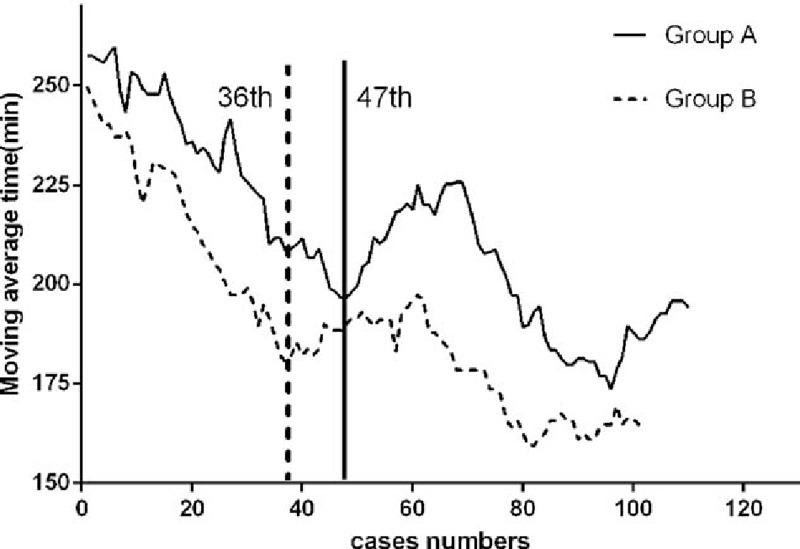
The moving average method shows that the operation time of both groups A and B decreased with increasing experience; and the first deflection was at around the 40th (47th in group A and 36th in group B) case.

**Figure 2:**
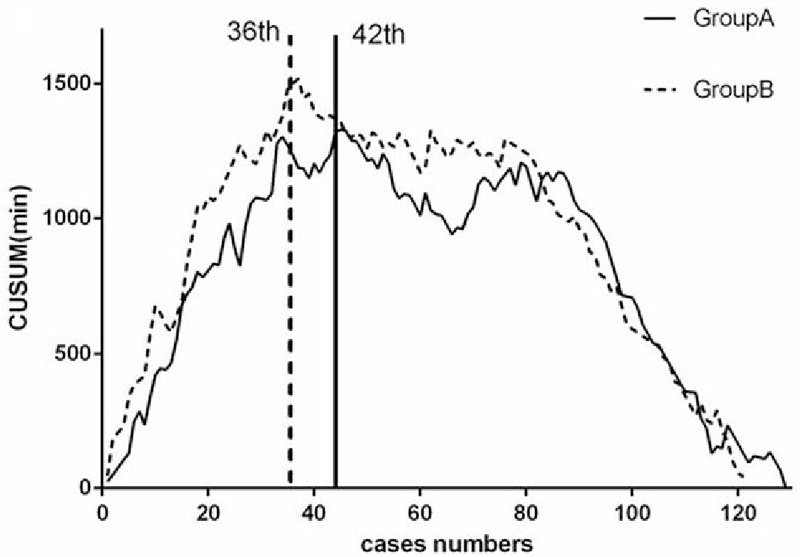
Cumulative sum analysis shows that the learning period of both groups A and B finished at around the 40th (42th in group A and 36th in group B) case.

**Figure 3:**
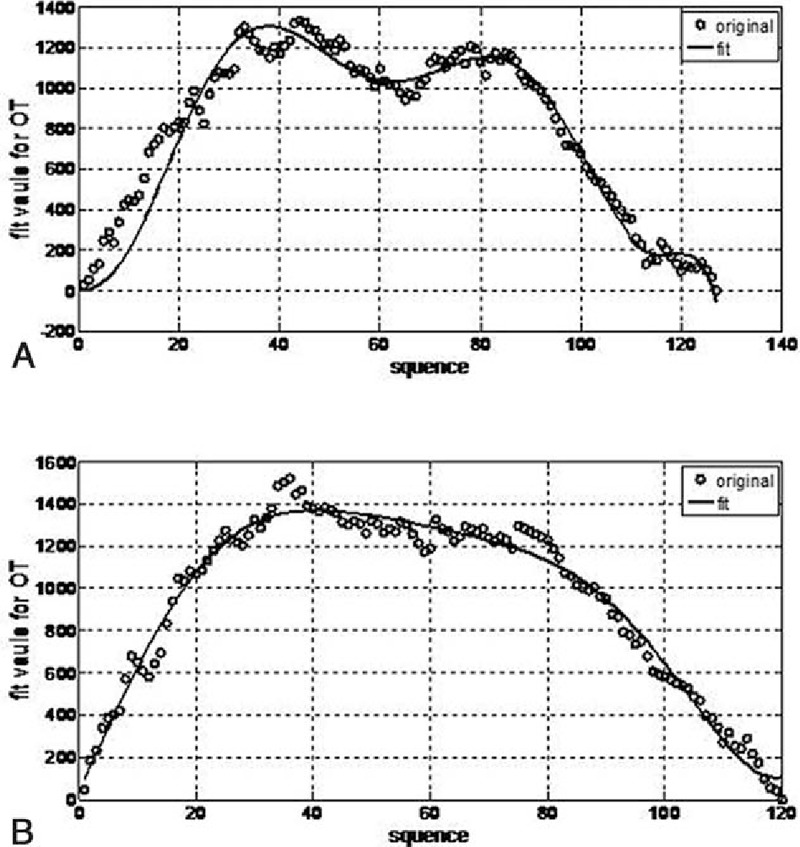
Matlab fitting curves of both groups A and B reached a plateau after the 40th case. (A) Group A and (B) group B.

In total, 119 patients underwent LAR in group A and 127 patients underwent LAR in group B. As shown in Fig. 1, the moving average method curve shows the overall trends for the operation time; the first inflection point was around the 40th (47th in group A and 36th in group B) case in the 2 groups. Similarly, CUSUM demonstrates that the peak points occurred at the 40th (42th in group A and 36th in group B) case in the 2 groups (Fig. 2). By fitting with Matlab software, the CUSUM curve shows that the peak point was at the 40th case (Fig. 3) in the 2 groups. Based on these findings, we concluded that the number of cases required to master the technique of LAR was 36 and 42 cases by group A and group B, respectively. So, in general, experience of operating on about 40 cases was required for surgeons to complete the learning phase.

### 3.2 Short- and long-term outcomes of LAR during the learning curve period

**Figure 4:**
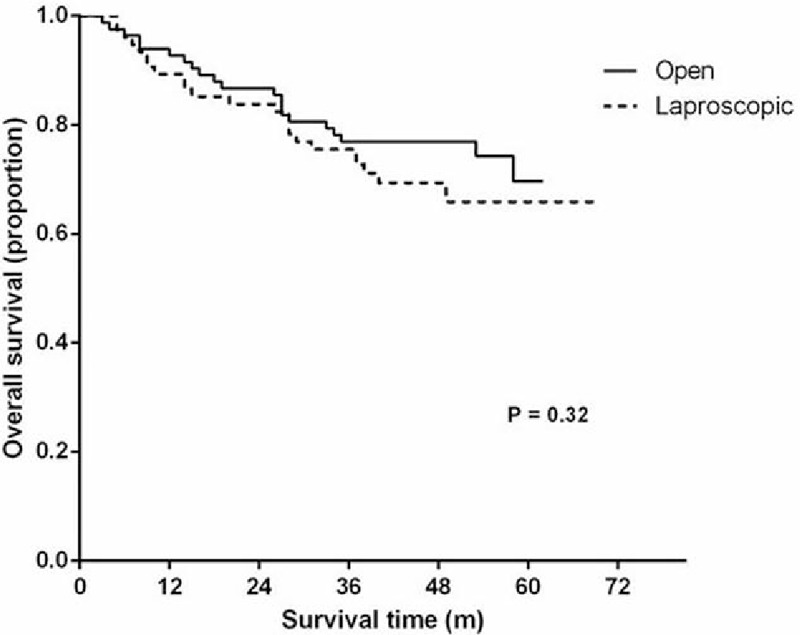
Kaplan–Meier curves comparing the 3-year overall survival rates of the laparoscopic-assisted rectal resection group with the open resection group operated during the learning period.

In this study, the surgeons in the 3 groups finished their 40th operation approximately by the end of 2012. Therefore, we regarded the patients operated during 2010 to 2012 as phase 1 (the learning curve period) and during 2013 to 2015 as phase 2 (the expert period). We collected data of 233 patients operated in phase 1, of which 112 underwent LAR and 121 underwent OR. The basic data were balanced as shown in Table 1, including age, sex, BMI, history of previous abdominal operation, accompanied disease, the average distance from the anal margin, ASA physical status classification, clinical stage (TNM) according to the American Joint Committee on Cancer guidelines (7th edition), tumor diameter, tumor histology, surgical procedures, prevented colostomy or ileostomy, lymphovascular invasion, and CEA level. Then, we compared the short- and long-term outcomes of LAR with OR operated by all 3 groups during the same period. Five (4.46%) patients in the LAR group required a transfer to the OR group. There were no differences in terms of the perioperative parameters between the LAR and OR groups. In addition, there were no statistically significant differences in the duration of postoperative hospital stay, lymph nodes harvested, urinary drainage time, time to first soft diet, distal margin, complications, or extra use of analgesic. Statistically significant differences in operation time, blood loss, time to first passage of flatus, proximal margin, and total cost were identified, and the operation time in the LAR group was longer than that in the OR group (Table 2). The follow-up rate in the LAR group was 67.86%, while it was 69.42% in the OR group. The estimated 3-year overall survival rates were similar between the 2 groups: 69.74% for the LAR group and 75% for the OR group (Fig. 4).

**Table 1:**
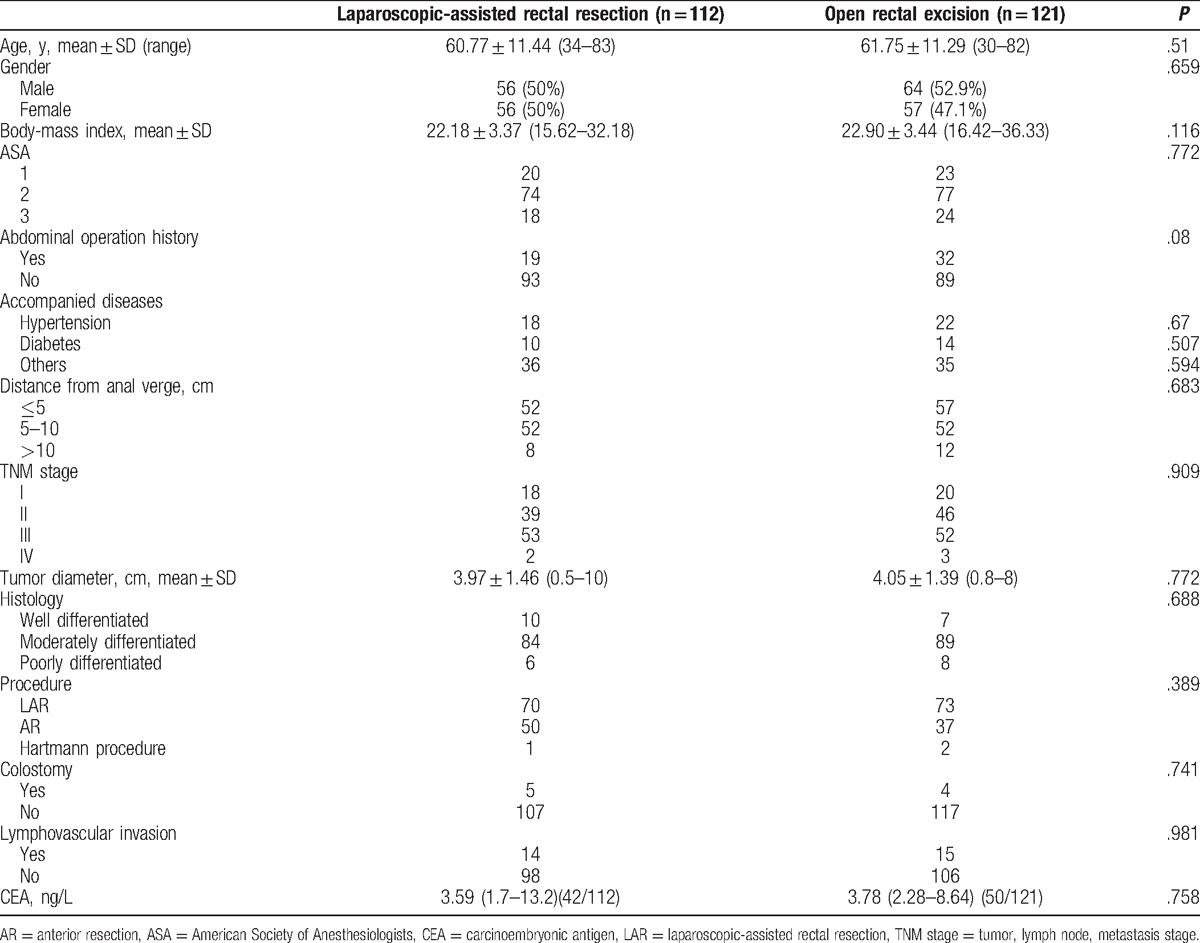
The basic data of 2 groups operated during the learning curve period.

**Table 2:**
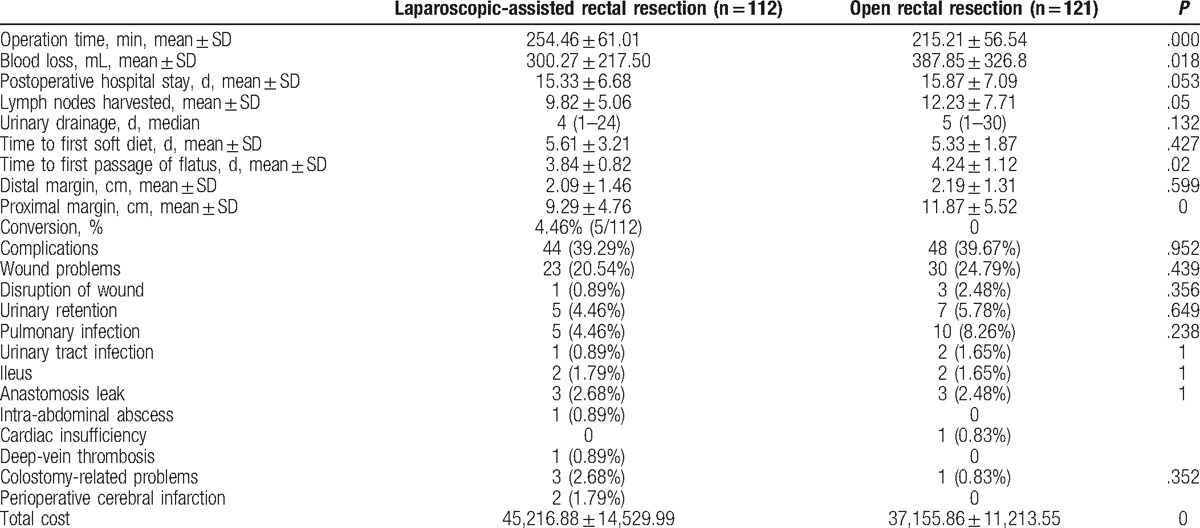
The short-term outcomes of 2 groups operated during the learning curve period.

### 3.3 Short-term outcomes of LAR in the expert period

To illustrate the short-term outcomes of LAR in the expert period, we collected 258 patients in phase 2: 169 patients underwent LAR and 89 underwent OR. The 2 groups were well balanced with regard to the perioperative parameters, including age, sex, BMI, abdominal operation history, accompanied disease, the average distance from the anal margin, ASA scores, TNM stage, tumor diameter, tumor histology, surgical procedures, colostomy or ileostomy, lymphovascular invasion, and CEA level (Table 3). As shown in Table 4, 2 (1.18%) patients in the LAR group required a transfer to the OR group. Laparoscopic surgery was associated with a significantly higher total cost (¥61,129.37 ± 15,072.30 vs ¥53,678.68 ± 14,728.23; *P* < .05), less estimated blood loss (86.33 ± 66.51 vs 271.34 ± 241.99 mL; *P* < .05), a shorter postoperative hospital stay (11.98 ± 3.17 days vs 13.29 ± 4.11 days; *P* < .05), a shorter urinary drainage time (4 days vs 6 days; *P* < .05), a shorter time to first soft diet (5.20 ± 2.05 days vs 5.87 ± 2.24 days; *P* < .05), a shorter time to first passage of flatus (3.84 ± 0.82 days vs 4.24 ± 1.12 days; *P* < .05), and a shorter proximal margin (8.28 ± 4.72 cm vs 12.35 ± 6.56 cm; *P* < .05), when compared with open surgery. The operation time, lymph nodes harvested, distal margin, complications, and extra use of analgesic were not significantly different between the LAR and OR groups.

**Table 3:**
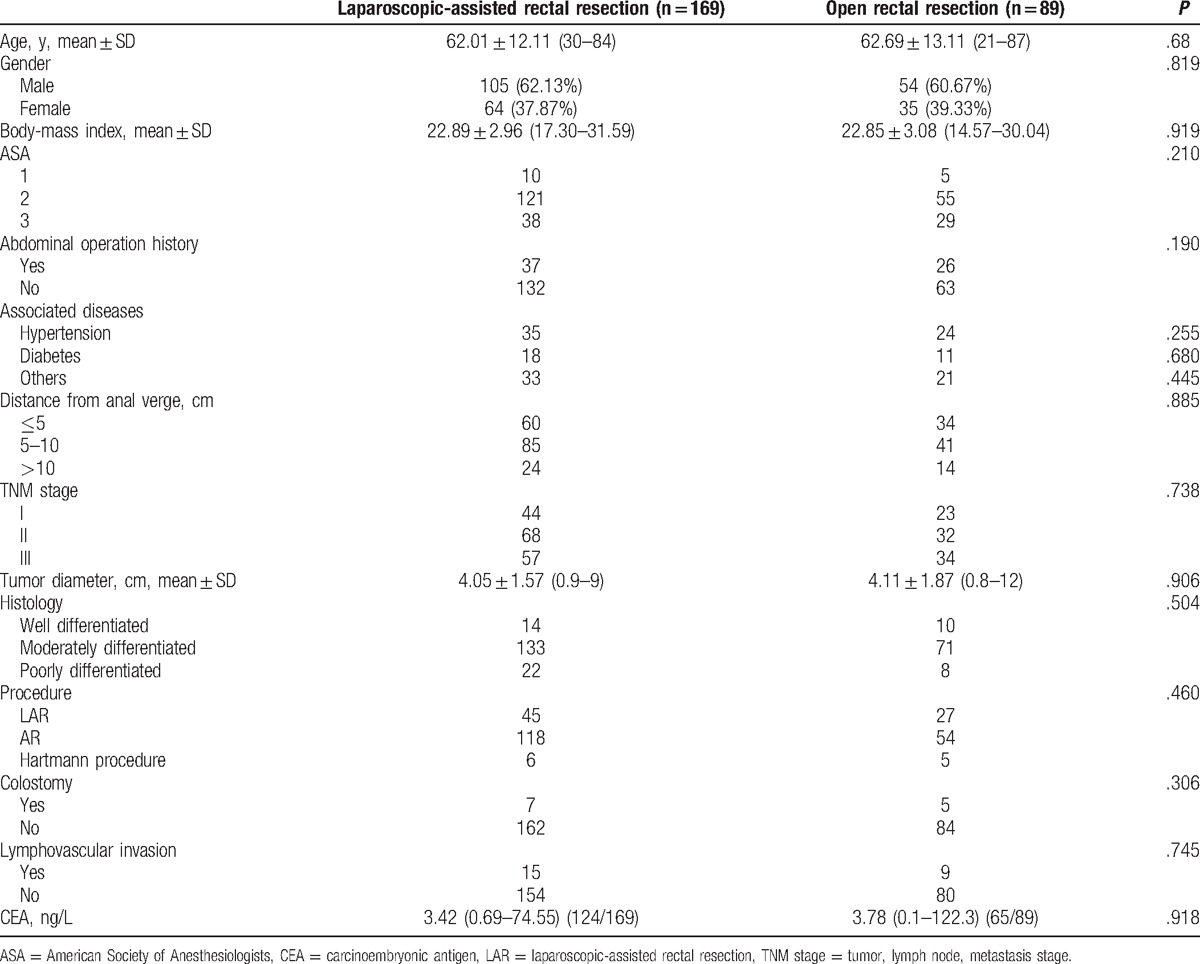
The basic data of 2 groups operated during the expert period.

**Table 4:**
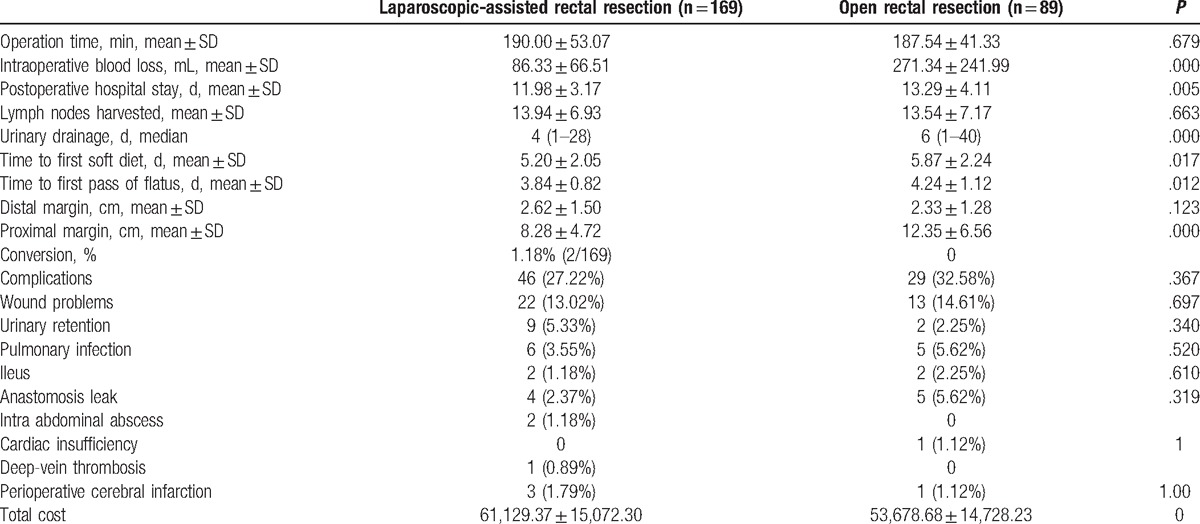
The short-term effects of the operation in 2 groups during the expert period.

## 4 Discussion

Since the first description of laparoscopic colectomy in 1991, multi-institutional randomized clinical trials have demonstrated its efficacy and safety.^[16–20]^ However, laparoscopic rectal resection remains controversial because of technical difficulty, including difficulties for pelvic exposure, sphincter preservation, and a steep learning curve.^[21,22]^ The learning curve reflects the learning process of a doctor for a certain type of surgery. Regrettably, the operations that take place during the learning process may not be perfect.

In order to determine the learning curve, we selected an appropriate outcome measure that indirectly acts as a proxy for the measurement of the ability of a surgeon to perform that particular task on a temporal basis. This outcome variable can be classified into 2 distinct categories: measures the patient outcome and quality assurance and measures the clinical procedure and task efficiency. The present study provided a multidimensional assessment of the learning curve for LAR via addressing multiple indicators of surgical performance. These included operation time, postoperative fast recovery, postoperative complications, switch to open surgery, and 3-year survival rates; the first parameter addresses the task efficiency and the latter 4 parameters measure the patient outcome as a surrogate marker of clinical effectiveness. In this study, we chose operation time to study our learning curve, which is the most acceptable parameter to analyze the curve.^[8,9,23,24]^

Here, we divided the patients into 2 phases according to the Matlab fitting curve and the peak point in the CUSUM graphs, and clinical and oncological outcomes were analyzed. The moving average method demonstrated the first deflection at around the 40th case. After 40 cases, the overall operation time became stable in both groups. In our study, the CUSUM graphs and the Matlab fitting curve also showed peak points at around the 40th case. Hence, we considered that the learning period was complete at around the 40th case. Beyond this stage, the doctors became familiar with the technique. We further found that all 3 groups finished their first 40 cases around the end of 2012. So, we included the operations completed before 2013 in the first phase (the learning curve period).

In previous studies, the rate of conversion to open surgery was used as a surrogate marker for the surgical capability.^[25]^ Both complications and conversion should be assessed for the learning curve.^[26]^ In our study, conversion seemed to occur more frequently in phase 1 (Table 2), possibly due to a poor surgical technique and experience. Then, we compared the short-term outcomes of the LAR and OR groups of the 2 periods mentioned above. During phase 1, we found that the short-term clinical outcomes of the LAR group were not superior to those of the OR group. The LAR group had a longer operation time in spite of less blood loss, but the postoperative recovery was similar in both the LAR and OR groups, including postoperative hospital stay, urinary drainage time, time to first soft diet, and time to first passage of flatus (Table 2). Longer postoperative hospital stay, time to drainage tube removal, and time to first soft diet in this study compared to previous reports^[18,19]^ were because of the patient's demand to stay longer against standard duration of hospitalization or the surgeon's desire for an extended postoperative observation. Also, the enhanced recovery after surgery protocol was not followed during the study period. The present study confirmed that there were no differences in lymph nodes harvested, distal margin, or postoperative complications between the LAR and OR groups in this phase.

The 3-year overall survival rate (69.74%) in the LAR group during phase 1 in the present study was comparable to a similar report that estimated it to be 73.7%.^[27]^ We found that there was no difference in the 3-year overall survival rate between the LAR and OR groups during phase 1. The present results were consistent with those findings in which LAR appeared to be equivalent to OR during the learning curve period.^[16,28]^ It also can be assumed that a postoperative fast recovery during the learning curve period may be compromised because of a surgeon's poor surgical skills during the initial training period, but the 3-year overall survival rates were not affected.

However, after we exceeded this period, the surgeons had mastered the laparoscopic technique, including laparoscopic ultra-low rectal resection. The operation time was shorter with less blood loss (Table 4). There was also a lower incidence of complications, although no statistical difference was found. Interestingly, the postoperative recovery of the LAR group was superior to that of the OR group in the expert period, including less estimated blood loss, a shorter postoperative hospital stay, a shorter urinary drainage time, a shorter time to first soft diet, and a shorter time to first passage of flatus. Lymph nodes that were harvested in the expert period, which reflected the oncological outcomes, were not significantly different between the LAR and OR groups.

One of the limitations of this study is that the number of patients was not enough to reach a definitive conclusion about oncological outcomes. Second, the present study was a retrospective analysis of the data. Prospective randomized controlled trials are required to validate the findings of this study.

## 5 Conclusion

The operation time and the postoperative recovery of LAR are not less than those of OR during the learning curve period (Table 2). However, LAR during the learning curve period showed similar clinical effectiveness and 3-year survival rates, compared to the OR group. These findings show that LAR is an acceptable method during the learning curve period. However, surgeons should take care and experienced surgeons should supervise surgical procedures to obtain satisfactory oncological outcomes in the learning curve period.
